# A Systematic Review of Pediatric Dialysis in Asia: Unveiling Demographic Trends, Clinical Representation, and Outcomes

**DOI:** 10.7759/cureus.51978

**Published:** 2024-01-09

**Authors:** Tanzir I Britto, Mohammad E Hoque, Shaikh A Fattah

**Affiliations:** 1 Endocrinology and Diabetes, Bangladesh Institute of Research and Rehabilitation in Diabetes, Endocrine and Metabolic Disorders, Dhaka, BGD; 2 Nephrology, Chittagong Medical College Hospital, Chittagong, BGD; 3 Nephrology, National Institute of Kidney Diseases and Urology, Dhaka, BGD; 4 Nephrology, Holy Family Hospital, Dhaka, BGD; 5 Medicine, Green Life Medical College, Dhaka, BGD

**Keywords:** end-stage renal disease, chronic kidney disease, acute kidney injury, systematic review, pediatric dialysis

## Abstract

Dialysis in pediatric groups is complicated by a wide range of factors that can affect long-term prognosis. The purpose of this meta-analysis and systematic review is to better understand the demographic and clinical factors that affect dialysis success in children. We searched a variety of databases for relevant articles and included 14 reports that dealt with the case studies of pediatric patients undergoing dialysis for a wide range of renal diseases. Patients' demographics, clinical presentations, laboratory findings, and treatment outcomes were the primary areas of data collection. To get a better sense of the overall prevalence of certain outcomes and to spot noteworthy trends or patterns in the disease process, we conducted a meta-analysis. Variations in dialysis efficacy and outcomes are highlighted throughout a wide range of ages in the pediatric dialysis cohort, from neonates to teenagers. Acute kidney injuries (AKI) tended to impact more boys, but chronic kidney diseases (CKD), such as lupus nephritis, disproportionately afflicted girls. Many different ethnic groups were represented, and there was evidence that some diseases having a hereditary component were more common in some areas than others. However, the potential for long-term consequences remained a concern. Hemodialysis was found to be effective in controlling end-stage renal disease (ESRD) and AKI, with some patients going on to have a kidney transplant. At the same time, peritoneal dialysis was associated with an increased risk of infection. This comprehensive analysis highlights the importance of demographic and clinical parameters in determining pediatric dialysis outcomes. A 14.47% mortality rate and gender disparities are revealed by this meta-analysis of pediatric renal diseases, which included a cohort of 235 patients with conditions like lupus nephritis and hepatitis C infection. The findings stress the necessity for individualized treatment techniques and suggest that demographic characteristics should be addressed in prognostic models. For better patient outcomes, the study also suggests standardized reporting in pediatric dialysis studies.

## Introduction and background

Pediatric dialysis, which is usually only used for a short time, is very important for children with long-term kidney problems [1]. Children who have acute kidney failure need dialysis to remove toxins from their blood and make up for the fact that their kidneys are not working as well as they should [1-3]. Children who need pediatric dialysis require special care that takes both their physical and emotional needs into account to make sure they stay healthy. Chronic kidney disease (CKD) can result in permanent kidney failure if left untreated, requiring a kidney transplant or lifelong dialysis [2]. In the same way, if acute kidney injuries (AKI) are not promptly and successfully treated, they may also result in significant kidney damage and comparable consequences [2]. In Asia, health care for children is important because the continent has a large, diverse population, and there are gaps in the quality and availability of health care [2]. Though public health improvements have changed the way childhood diseases are thought of in Asia, pediatric nephrology faces new problems that need to be fixed to protect the health and future of many children [2]. Research shows that CKD is an important issue in Asia [3]. It may be even worse in some Asian countries, like China and India, than in other places, like North America and Europe [3]. The prevalence of CKD is much higher in Asia, where Up to 434 million people in Eastern, Southern, and South-Eastern Asia have CKD, with 65 million having more advanced types, according to the study [3, 4], but most of the studies that have been done on CKD is within adult and studies done with children have been done with non-Asian countries children [5]. Children and adolescents face different kidney disease management and treatment challenges than adults. Documenting pediatric dialysis case studies may help doctors and researchers share their real-life discoveries [6, 7]. Healthcare professionals can better understand pediatric renal patients and their treatment by discussing difficult instances [6]. Doctors can promote innovative technologies and therapeutic approaches to better pediatric renal disease therapy [6]. Case studies in pediatric dialysis can help identify sickness patterns and trends, improving treatment outcomes. Case studies can also demonstrate how regional clinical practices, healthcare infrastructures, genetics, and environment affect pediatric dialysis management. 

The objective of this review paper is to comprehensively analyze case studies on pediatric dialysis in Asia, sourced from the digital libraries Google Scholar and PubMed, spanning the years January 2000 to October 2023. We also searched Google to find more case studies. The aim is to gain insights into rare complications, presentations, and outcomes that can enhance clinical decision-making in uncommon scenarios.

The later part of the introduction briefly describes the background of dialysis and pediatric dialysis, describing their clinical characteristics worldwide. In the review section, a review of the situation in Asia regarding pediatric dialysis was discussed. Finally, the last section concludes the paper by highlighting the main findings of the papers. 

Background

Generally, end-stage renal disease (ESRD) patients require renal replacement therapy (RRT) like dialysis or transplantation to survive, with options including hemofiltration, hemodiafiltration, and both hemodialysis and peritoneal dialysis [8]. Kidney transplants offer a more permanent solution, replacing the damaged kidney with a donor kidney [8]. These therapies are critical for ESRD patients, whose condition represents the final stage of CKD and AKI, where kidney function is nearly lost, necessitating artificial methods to perform the kidneys' filtering roles [9]. However, these life-supporting treatments come with infection risks due to the compromised immune systems of those with established renal failure (ERF) [8-10]. Children, from birth up to 18 years of age, typically undergo peritoneal dialysis at home or hemodialysis to remove blood waste products, with the former using the abdomen's peritoneum as a filter and the latter using a dialyzer machine [11]. While hemodialysis is often done in clinics, home options are available [11]. Both are critical for children with ESRD to perform the detoxification roles of failed kidneys [11].

The primary reason for pediatric dialysis is ESRD, caused by various conditions like severe electrolyte imbalance, acute kidney injuries due to trauma, or chronic diseases hypertension [12]. Glomerular diseases like nephrotic syndrome and focal segmental glomerulosclerosis (FSGS) also lead to ESRD in children [12]. Differences in CKD prevalence worldwide may be influenced by racial diversity, screening practices, and healthcare access disparities, with a significant burden noted in Asia due to varying levels of healthcare infrastructure and economic development [3,9,13]. CKD epidemiology varies by country and is influenced by racial differences, screening practices, and educational programs. In Asia, since the first pediatric nephrology group in 1986, access to dialysis has improved [2], particularly in developed areas like Japan, Taiwan, and Korea, where 0.1-0.2% of the population are on dialysis [13]. While Western countries have been the focus of many CKD and AKI studies in children, Asian-specific research, especially in Japan, has highlighted the importance of early intervention for CKD stages 3-5. Asia offers a range of dialysis treatments for children, reflecting significant advancements in pediatric kidney care [2, 9]. 

## Review

Search method

This review paper used the Preferred Reporting Items for Systematic Review and Meta-Analysis (PRISMA) 2020 standards as a framework for conducting the systematic review. We conducted a comprehensive search on October 10, 2023, in two electronic databases, namely Google Scholar and PubMed, and we also searched in Google, covering the period from January 2000 to October 2023. We used the keywords "pediatric dialysis", "case study", and "Asia".

Search criteria

This review paper is a secondary study that exclusively used data from previously published sources and did not involve any patients in its formulation or execution. The research question was established and developed, and the study was conducted without direct patient contribution. Additionally, there are no plans to involve patients in the dissemination of this study's findings. The focus of the research was on reviewing case studies about pediatric dialysis within Asian populations. This study covers Asian research on patients under 19, so we excluded adult dialysis case studies from our analysis. Non-English papers were omitted. Early on, 51 papers were collected from Google Scholar, 20 from PubMed, and 10 from a Google search. We eliminated duplicates and non-English documents on both ends. By examining titles and abstracts, non-English articles were excluded. Our research focused on Asian pediatric dialysis.

Inclusion and Exclusion Criteria

Open-access pediatric dialysis case studies done in the Asia region were included in this study. Non-English studies, adult dialysis, non-Asia studies, and articles that were not free access were excluded.

**Figure 1:**
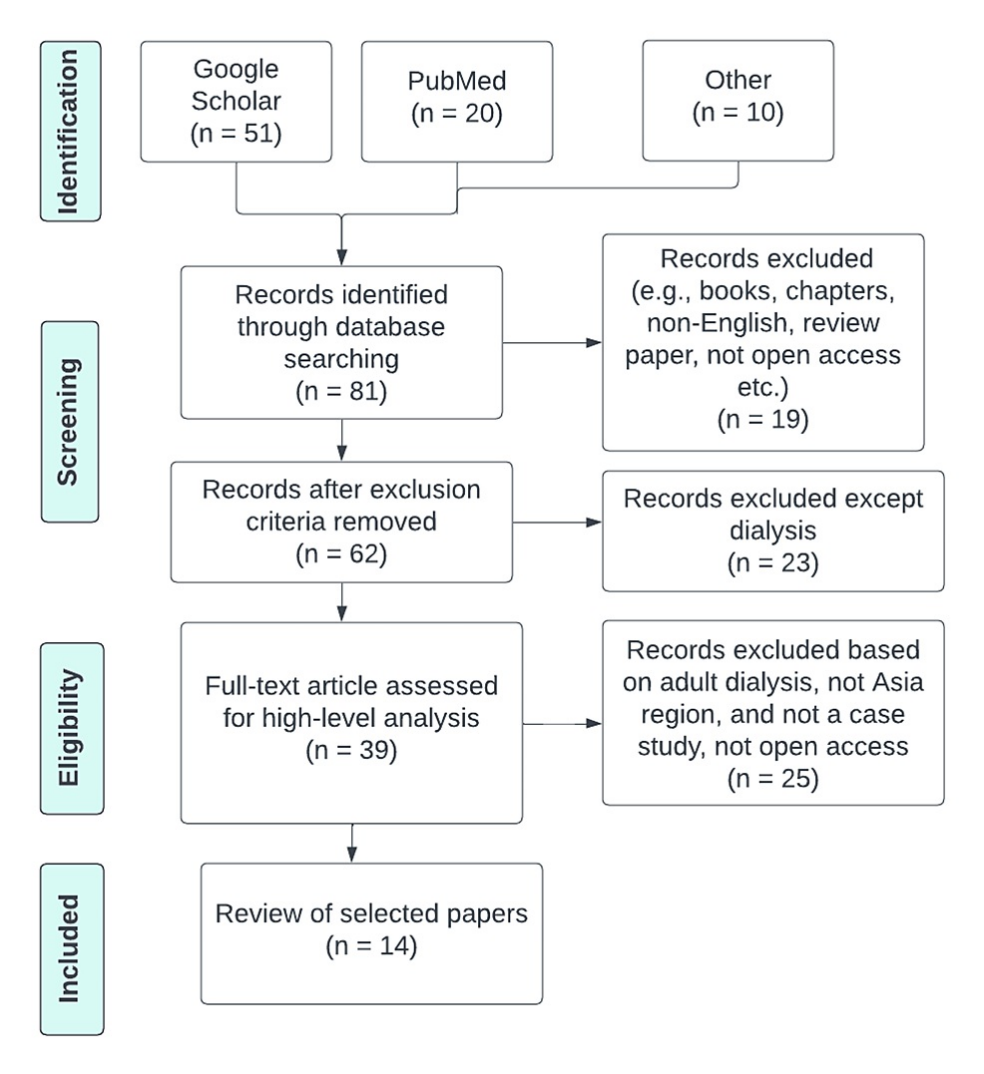
Preferred Reporting Items for Systematic Reviews and Meta-Analyses (PRISMA) flow chart of research conducted on pediatric dialysis in Asia, 2000-2023

In total, 14 publications were obtained from the aforementioned investigations. Figure 1 shows the step-by-step procedure that has been followed using PRISMA. The flowchart shows the systematic review paper screening and selection process.

Results

Demographic Characteristics of Pediatric Dialysis Patients

There are 14 reports included in the aggregate data, and the underlying diseases that required dialysis vary significantly. The youngest patient in our analysis is a four-day-old female with renal tubular dysgenesis (RTD), highlighting the potential need for immediate dialysis post-birth [14]. Teenagers in their early 14s are the eldest patients, with diseases including lupus nephritis [15] and hepatitis C following kidney transplantation [16] requiring treatment.

Overall, the case studies show a fairly even gender split, with some acute illnesses, like AKI in neonates, having a slight male predominance [17]. On the other hand, females tend to be more affected by childhood-onset lupus nephritis [15]. Consanguinity is also revealed by demographic research, especially in RTD [14] and rhabdomyolysis related to LPIN1(Phosphatidate phosphatase-1 (lipin-1) efficiency [18], which may indicate a genetic susceptibility and a higher incidence in certain regions or groups.

Chinese [15, 19], Indonesian [16, 20], Kazakh [21], Turkish [14, 18], Japanese [22], Qatari [23], Nepalese [24], Indian [25, 17], and Bangladeshi [26, 27] pediatric patients are all included in the dataset. This range emphasizes how important it is to have culturally and regionally appropriate medical procedures. In certain situations, the gestational age and birth weight were disclosed. It is noteworthy that the majority of newborns with AKI had birth weights larger than 2500 grams, given that low birth weight is a risk factor for kidney problems [27].

Clinical Presentations and Measurements

The symptoms varied widely in their clinical presentation, from respiratory distress and anuria [14] to severe sepsis [21] and recurring rhabdomyolysis [18]. Laboratory results, including blood urea nitrogen, serum creatinine, and inflammatory markers, were given, along with measurements like blood pressure and pulse rate. As an example, the renal tubular dysgenesis case [14] brought to light important clinical parameters such as hyperkalemia and a blood pressure of 64/22 mmHg.

Laboratory and Histopathological Findings

The results of laboratory tests played a critical role in the diagnosis and evaluation of disease severity. Elevated blood creatinine and hypoalbuminemia are common symptoms in cases of acute kidney injury (AKI) and nephrotic syndrome [17, 19]. Serum creatine kinase (CK) values in a patient with the LPIN1 gene deficiency [18] were remarkably elevated, reaching 943,452 IU/L. In certain cases, such as RTD with underdeveloped proximal tubules [14] and nephrotic syndrome with proliferative sclerosing glomerulonephritis [19], histopathological abnormalities were reported.

Efficacy of Treatment Interventions and Dialysis Outcomes in Pediatric Renal Diseases

This section includes a thorough study of the effectiveness of dialysis as well as a review of various treatment approaches and their results in pediatric renal diseases. Various degrees of success were observed in terms of outcomes and therapeutic efficacy in interventions such as hemoadsorption in pediatric septic patients [21] and direct-acting antiviral agents (DAAs) in adolescents with hepatitis C and kidney transplantation [16]. There have also been reports on the long-term prognosis for individuals with childhood-onset lupus nephritis [15]. A more comprehensive view of the patient populations, their clinical characteristics, demographics, and specific laboratory results related to the illnesses investigated in Asia can be obtained from our study's Tables 1 and 2. Additionally, we investigate the use of dialysis in the treatment of pediatric renal diseases. These treatments had a variety of effects, from long-term survival with intact renal function to mortality owing to comorbidity [14, 19, 25].

Notably, one patient who underwent peritoneal dialysis later died from sepsis [14], highlighting the risk of infection with this treatment. However, hemodialysis, both peritoneal and conventional, has been instrumental in stabilizing patients with end-stage renal disease (ESRD) and acute kidney injury (AKI), leading to successful renal transplantation in some cases [19, 21, 16]. An improvement in the survival rate of juvenile patients with AKI from 69% to 71% was observed following dialysis [24], demonstrating its importance in treatment, albeit with some patients still facing poor outcomes. This highlights the complex nature of pediatric dialysis, balancing acute condition management and long-term sequelae prevention.

Table 1 illustrates the demographics, clinical characteristics, and laboratory findings to provide a clearer picture of the patient populations and their respective outcomes in Asia. Table 2 describes the detailed laboratory findings associated with the conditions studied.

**Table 1:** Demographic and clinical characteristics of pediatric patients CKD - chronic kidney disease; AKI - acute kidney injuries; BP - blood pressure; HCV - hepatitis C virus; HAV - hepatitis A virus; RNA - ribonucleic acid; NEC - necrotizing enterocolitis; LPN1 - lipin-1 gene; CK - creatinine kinase; AST - aspartate aminotransferase; ALT - alanine aminotransferase; MODS - multiple organ dysfunction syndrome; NOMID - neonatal-onset multisystem inflammatory disease; CINCA - chronic infantile neurological cutaneous and articular; CRP - C-reactive protein; ESR - erythrocyte sedimentation rate; IL-6 - interleukin 6; PUV - posterior urethral valve; eGRF = estimated glomerular filtration rate; pPRIFLE - pediatric Risk, Injury, Failure, Loss, End-Stage Renal Disease; DAMA - discharge against medical advice

Ref.	Disease of focus	Age/gender	Clinical presentation	Key measurements and clinical notes	Outcome
Atasay et al. [14]	Renal tubular dysgenesis (RTD)	N=1; fout-day-old female	Anuria, respiratory distress, physical anomalies	BP: 64/22 mm of Hg, Pulse: 138/min, Temp: 36.2°C	Expired
Chan et al. [15]	Childhood-onset lupus nephritis (cLN)	N=92; 13.7 ± 3.3 years (78 female)	-	Assessment of kidney survival rates and CKD incidence	Expired: 2; ESRD: 3; Advanced CKD: 5; Discharged: 82
Ambarsari et al. [16]	Hepatitis C virus infection	N=2; 13 years (male)& 14 years (female)	ESRD due to various causes of HAV and HCV	Elevated liver enzymes, high HCV RNA levels	Discharged
Mishra et al. [17]	Acute kidney injury (AKI) in newborns	N=74; Newborns, (13 female)	Sepsis, NEC, low Apgar scores	High serum creatinine (>1.5 mg/dl)	Discharged: 21, DAMA: 38; Expired: 15
Topal et al. [18]	LPIN1 deficiency (rhabdomyolysis)	N=1; 26-month-old female	Appetite loss, somnolence, fever, dark urine	Elevated serum CK, AST, ALT levels	Discharged
Li et al. [19]	Steroid-resistant nephrotic syndrome	N=1; 8-year-old female	Foamy urine, edema, moon face, buffalo hump, hairiness	Weight: 26 kg, BP: 130/100 mmHg	Discharged; Kidney transplant after 2 years
Ambarsari et al. [20]	AKI and MODS due to wasp stings	N=2; 12 years (male) & 15 years (female)	-	-	Discharged: the female need renal replacement
Saparov et al. [21]	Sepsis	N=1; 8-month-old (gender not revealed)	Laryngeal stenosis, malnutrition, pneumonia, sepsis	BP dropped to 42/23 mmHg	Discharged
Kawashima et al. [22]	NOMID/CINCA syndrome	N=1; 11-year-old male	Fever, rash, craniofacial anomalies, ventricular enlargement	High CRP, ESR, IL-6, altered immunoglobulins	Condition remained challenging to manage
Mohammed et al. [23]	PUV with kidney dysplasia	N=1; 2-year-old male	History of PUV resection, recurrent peritonitis	Recurrent peritonitis, anemia	Discharged
Baranwal et al. [24]	Hemolytic uremic syndrome (D+HUS)	N=13; (female 4) 1-8 years, median 20 months	Dysentery, oligo-anuria, neurological issues	Various blood chemistry imbalances	Discharged: 9 Died: 3 DAMA: 1
Beeregowda et al. [25]	Formalin poisoning	N=1; 6-month-old female	Sensorium alteration, convulsions, hypertonia	Metabolic acidosis with high anion gap	Expired
Sultana et al. [26]	Frasier syndrome	N=1; 4-year-old female	Generalized edema, respiratory distress, high creatinine	Creatinine: 13.4 mg/dl, eGFR: 11 ml/min/1.73m²	Discharge with dialysis
Afroz et al. [27]	Neonatal acute kidney injury	N=44; (female 23) to 28 days old (Majority <7 days)	Majority neonates presented in the first week of life	Use of pRIFLE criteria for AKI diagnosis	Discharged: 32 Death: 12

**Table 2:** Summary of key laboratory findings across different conditions BUN - blood urea nitrogen; eGRF - estimated glomerular filtration rate; CKD - chronic kidney disease; CK - creatinine kinase; AST - aspartate aminotransferase; ALT - alanine aminotransferase; CRP - C-reactive protein; ESR - erythrocyte sedimentation rate; pRIFLE - pediatric Risk, Injury, Failure, Loss, End-Stage Renal Disease; AKI - acute kidney injury; IL-6 - interleukin 6

Key laboratory findings
Increased BUN/creatinine, metabolic acidosis, hyperkalemia, hyperuricemia, immature proximal tubules [14]
Assessment of eGFR for CKD progression [15]
Elevated liver enzymes pre and post-antiviral treatment [16]
High serum creatinine levels [17]
Extremely high CK, AST, ALT levels; myoglobinuria [18]
Proteinuria, hypoalbuminemia, hypercholesterolemia, reduced GFR
Hyperleukocytosis, high CRP and procalcitonin, elevated liver enzymes [21]
High CRP, ESR, IL-6, altered hemoglobulin and immunoglobulins [22]
Recurrent peritonitis, worsening anemia [23]
Anemia, thrombocytopenia, leukocytosis, varied electrolyte imbalance [24]
Elevated liver enzymes, high serum creatinine and urea, metabolic acidosis, high formic acid [25]
Hypercholesterolemia, hypoalbuminemia, nephrotic range proteinuria [26]
Decreased urine output as per pRIFLE criteria, various stages of AKI [27]

Diverse Spectrum and Outcomes of Pediatric Renal Diseases: A Comprehensive Meta-Analysis

The studies range from individual case reports to larger cohorts, covering a variety of conditions, including renal tubular dysgenesis, lupus nephritis, hepatitis C virus infection, acute kidney injury, and more.

This meta-analysis includes a variety of illnesses related to pediatric renal diseases, such as hepatitis C infection, renal tubular dysgenesis, and childhood-onset lupus nephritis. Patients range in age from newborns to early teenagers, and for some disorders, there are noticeable gender differences in the patient population. The symptoms that manifest clinically range from anuria and respiratory distress to fever and neurological problems. Serum creatinine, liver enzymes, and blood pressure are important metrics. Results can include the patient's death or their release with unresolved medical problems or the requirement for continuous care. With 235 patients in the sample as a whole, the mortality rate was roughly 14.47%. In conditions like lupus nephritis, the distribution of genders was biased towards women. Gender information was given for 234 participants in the pooled analysis of the studies, covering a total of 235 people. There were 96 men and 138 women among them. This indicates that 41.03% of patients are male and 58.97% of patients are female. This cohort's gender representation is comparatively balanced, which facilitates a more thorough assessment of the disease outcomes and any potential gender variances. These demographic insights are essential for understanding gender-based disparities in illness development and therapy response, as well as for customizing therapeutic methods for individual patients. This analysis highlights the need for more research and targeted medicines in pediatric renal disorders, emphasizing the significance of early diagnosis and customized therapy.

Synthesized Overview of Pediatric Dialysis: Challenges and Advancements

Pediatric dialysis patients require a variety of approaches to care that take into consideration their special physiological and developmental demands. Although survival rates have significantly increased because of breakthroughs in treatment tactics, these young children continue to confront significant and ongoing chronic problems. Numerous research and case reports that illustrate the changing field of pediatric nephrology-from the treatment of complicated genetic abnormalities to the acute onset of kidney injuries-can be found in the current literature. To maximize results in pediatric dialysis, this overview summarizes current studies that emphasize the significance of early intervention, the integration of innovative treatments, and the careful management of comorbid diseases.

Recent research has provided clarity on the complicated terrain of pediatric dialysis, highlighting both tremendous treatment progress and a variety of enduring difficulties. The results of multiple investigations are integrated into this synthesis, highlighting the complexity of pediatric nephrology.

Diverse pathologies and early intervention: Early dialysis intervention is crucial due to the variety of renal diseases that affect children, ranging from congenital abnormalities like renal tubular dysgenesis (RTD) [14] to acute kidney injury (AKI) in infants linked to sepsis and male gender [17]. These investigations emphasize the necessity of early and efficient intervention for a range of pediatric kidney disorders.

Chronic challenges and holistic management: Chronic problems still exist even with breakthroughs in medicine, as demonstrated by the treatment of pediatric lupus nephritis patients [15]. This study shows the value of treating patients holistically, with an emphasis on managing comorbid conditions such as hypertension and bone health.

Innovations in treatment and patient outcomes: New approaches to treatment are showing promise in improving patient outcomes. One such approach is the use of direct-acting antiviral medications in adolescents who have had kidney transplants [16]. Pediatric dialysis is a dynamic field, as evidenced by this evolution of treatment.

Genetic insights and personalized approaches: Novel gene mutations (LPIN1 [18] and WT1 [19]) have been found, and this has provided important information about the genetics of renal disorders. These results are especially important for genetic counseling and the development of tailored treatment plans for diseases that lead to dialysis-dependent ESRD.

Environmental factors and emergency responses: Environmental factors emphasize the necessity for early dialysis intervention in emergency settings, such as multiple wasp stings causing AKI and multiple organ failure syndrome [20]. These instances highlight how important dialysis is for treating sudden systemic responses.

Advances in dialysis techniques: Dialysis innovations such as continuous hemodiafiltration for disorders like NOMID [22] or hemoadsorption with CytoSorb® (CytoSorb, Berlin, Germany) for severe sepsis [21] demonstrate the adaptability and efficacy of contemporary dialysis procedures in treating difficult pediatric conditions.

Diagnostic complexities and care management: The diagnostic challenges in pediatric dialysis are exemplified by cases such as recurrent peritonitis in a child receiving peritoneal dialysis, which was caused by an ingested foreign body [23]. Furthermore, the treatment of illnesses such as hemolytic uremic syndrome coupled with diarrhea (D+HUS) [24] emphasizes the advantages of specialized care in raising the survival rates of pediatric dialysis patients.

Acute kidney injury in newborns: The issue of AKI in neonates was clarified by Mishra et al. (2022) [17], who found strong correlations between sepsis and male gender. The significance of timely identification and treatment in enhancing prognosis and mitigating mortality risks in this susceptible population is underscored by their findings.

As these studies [14-24] demonstrate, the field of pediatric dialysis is changing, necessitating an integrated approach that includes early intervention, adaptation of new medications, and comprehensive care of concomitant diseases. This approach is vital to enhance outcomes and improve the quality of life for pediatric patients on dialysis.

Discussion

The provision of pediatric dialysis in Asia presents a unique landscape shaped by diverse demographics, intricate genetic predispositions, and variable socio-economic factors. This review, synthesized through a PRISMA-guided approach, has amalgamated a wealth of case studies that underscore the complexity and the nuanced challenges of managing pediatric renal failure within this vast continent.

The demographic variability of this patient cohort is significant. The wide age spectrum from neonates to adolescents [14, 15, 24, 27], the roughly equivalent gender distribution [14, 19, 21, 22], and the varied ethnic backgrounds reflect the extensive scope clinicians must consider when devising treatment plans. Interestingly, a male predominance in neonatal acute kidney injury [17, 27] hints at a gender-related susceptibility that may require further exploration to understand the underlying biological mechanisms. The impact of genetic factors is evident, with consanguinity playing a role in conditions like renal tubular dysgenesis [14] and LPIN1-related rhabdomyolysis [18]. This underlines the importance of genetic screening and counseling, particularly in regions where consanguineous marriages are more prevalent. Early identification of genetic disorders and prompt initiation of dialysis can be life-saving, especially in critical early-life presentations [14].

The variability in presentation ages also dictates differing management strategies. For instance, older children with lupus nephritis [15] face chronic disease issues such as transition to adult services and long-term immunosuppression management, contrasting sharply with the immediate life-threatening issues seen in neonatal presentations [14].

Geographical and socio-economic differences within Asia have profound implications for dialysis access and management. Rural settings may encounter environmental nephrotoxins, as seen with formalin poisoning [25], while urban areas might have better healthcare infrastructures to manage complex conditions like hepatitis C post-transplantation [16]. The diverse range of settings within the continent indicates the necessity for adaptable healthcare strategies that can serve varied local needs.

Recent advancements in treatment are encouraging, particularly in lupus nephritis, where improved immunosuppressive regimens are enhancing long-term outcomes [15]. However, this is contrasted by enduring challenges in managing complications of dialysis, such as peritonitis [23] and the need for emergent interventions in conditions precipitating acute kidney injury [19, 17]. The role of genetics extends beyond inherited conditions; it also encompasses a need for tailored pharmacogenomic approaches. The cases highlighting novel genetic mutations [18, 26] demonstrate the ever-expanding knowledge of disease etiology, which could potentially pave the way for personalized medicine in pediatric nephrology.

The innovation in therapeutic modalities, such as the use of CytoSorb® in septic patients [21], represents the cutting edge of pediatric critical care nephrology. These advances show promise for improving outcomes in children with renal failure complicated by systemic illnesses.

This review reaffirms the necessity for ongoing research into pediatric dialysis, particularly in a geographically and ethnically diverse setting like Asia. While the challenges are daunting, ranging from genetic disorders to environmental exposures leading to acute kidney injury, the potential for improving pediatric nephrology care is vast. Emerging data suggest that long-term patient monitoring, advancements in dialysis techniques, and personalized care plans are essential to improve survival and quality of life for these young patients. A graphical representation of the progression and outcomes of dialysis in these patients would be a valuable tool for clinicians and researchers alike, providing a clearer picture of the disease trajectories.

## Conclusions

In conclusion, pediatric dialysis care in Asia requires a concerted effort to adapt to its demographic and socio-economic diversity. The cases discussed herein illustrate the necessity for meticulous and culturally sensitive approaches to treatment, comprehensive genetic counseling, and a commitment to technological and therapeutic innovation to address the myriad of challenges presented by pediatric renal failure. The study primarily focuses on pediatric dialysis within the Asian context, presenting a limited scope that may not fully represent global perspectives. This regional concentration restricts the generalizability of the findings to broader populations. Furthermore, the variability in methodologies across the included studies introduces potential inconsistencies in data interpretation, challenging the uniformity of the conclusions drawn. Additionally, the lack of a comparative analysis with non-Asian data limits the depth of understanding regarding regional differences in pediatric dialysis outcomes and practices. In further studies of pediatric dialysis, we plan to extend our research beyond the Asian context to gain a more global perspective, and we will use additional search engines to acquire more comprehensive information on this topic. This expansion will enhance the generalizability of findings and provide insights into various regional practices and outcomes. Additionally, standardizing methodologies across studies could reduce data inconsistencies, leading to more robust and uniform conclusions. A comparative analysis with non-Asian data should also be incorporated to deepen the understanding of regional differences in pediatric dialysis care.
